# The Role of Fundamental Nursing Practices Simulation on Students’ Competencies and Learning Satisfaction: Repeated Measured Design

**DOI:** 10.3390/healthcare10050841

**Published:** 2022-05-03

**Authors:** Gizell Green, Lani Ofri, Riki Tesler

**Affiliations:** 1 Department of Nursing, Faculty of Health Science, Ariel University, Ariel 40700, Israel; laniof@ariel.ac.il; 2 Department of Health Systems Management, School of Health Sciences, Ariel University, Ariel 40700, Israel

**Keywords:** nursing students, simulation, nursing competencies, learning satisfaction

## Abstract

Few studies have examined the simulation of fundamental nursing practices regarding nursing competencies and learning satisfaction via repeated measured methods. Objectives: To evaluate a simulation of fundamental nursing practices on nursing students’ competencies and learning satisfaction in three time points: before (T1), immediately after (T2), and one month after simulation (T3), and to examine nursing students’ competency predictors to learning satisfaction, immediately after conducting the simulation and one month after. Methods: The study design was a one-group, repeated measures study. Ninety-three undergraduate nursing students were convenience sampled and conducted a simulation of fundamental nursing practices. The students completed a questionnaire at T1, T2, and T3. The Competency Inventory for Registered Nurses questionnaire was distributed, and question about the level of learning satisfaction were asked. Results: All nursing competencies and learning satisfactions increased significantly. Only the legal/ethical practice competency succeeded in predicting the learning satisfaction in T2 and in T3 after conducting simulations. Conclusions: This study has established that a simulation of fundamental nursing practices is effective not just immediately after performing the simulation but also one month after the simulation. Therefore, it is recommended to implement a pedagogical structure of simulations of fundamental nursing practices in other nursing education areas.

## 1. Introduction

The complexity of the current healthcare environment poses challenges to healthcare providers such as nurses, whose knowledge and skills are evolving. Nursing educators need to be able to provide opportunities for their students to practice high order thinking skills, though an insufficient number of appropriate clinical placements limit students’ experiences with real patients. Simulated clinical training experiences can provide an essentially risk-free approach to learning in a setting close to authenticity while allowing students to construct knowledge and develop skills in a safe setting [1].

Simulation-based learning plays an important role in helping students and experts conduct clinical exercises, as it provides a chance for the replication of clinical performance [2]. Simulation-based learning is described as constructing an artificial condition that represents the experience of a clinical environment, in which students can practice and build their experience [3]. One study recommended integrating virtual simulations when used in conjunction with manikin-based simulations and assessing their contribution [4]. A meta-analysis study demonstrated that simulation programs were more beneficial than traditional learning methods [5]. In addition, nursing simulation training has been found to increase thinking capabilities [6,7,8], such as critical thinking [9], which is vital for nurses. It contains the ability to prepare students for the clinical environment by allowing them to practice in a safe environment and in complex clinical circumstances to improve clinical knowledge. Simulation is another way that enables the evaluation of students’ clinical skills, and thus provides support for their learning in nursing teaching programs [10,11]. Additionally, simulation is a practicable strategy to assess students’ clinical competency in nursing programs [10].

Nursing competence refers to the skills required to accomplish the role of nurses [12]. The areas of nursing competence are diverse, including knowledge, skills, values, attitudes, communication, collaboration abilities, critical thinking, and innovation [13]. In this study, we chose to focus on the following nursing competencies: legal/ethical practice, interpersonal relations, leadership, professional development, clinical care, critical thinking/research aptitude, and teaching/coaching, since they were based on the International Council of Nurses Framework of Competencies for the Generalist Nurse [14]. In order to carry out nursing responsibilities appropriately, nursing simulation learning contributes to the competency of nursing students, requiring them to integrate knowledge, skills, communication, critical thinking, and reflection into the critical thinking process [15,16]. The use of simulation technologies enables nursing students to apply knowledge and skills to move from theory into practice and contributes to improving long-term clinical knowledge. Moreover, the practice in the simulation helps improve the capability of explaining problems among nurses and patients, which strengthens thinking skills by analyzing nursing problems from different aspects for the process of problem-solving [17], which is considered a highly important nursing competence. There are other positive aspects of simultaneous intervention, one of which is student satisfaction.

Overall, the satisfaction of nursing students regarding the practice of nursing skills in the simulation is varied [18,19,20,21]. It has been found that there is a high level in the safety feelings of the students who practiced simulation, as it simulated conditions that were very close to the clinical field, and they gained satisfaction from clinical experience [18]. Simulation-based learning has been found to be effective in improving students’ perceived competence in nursing and learning satisfaction.

The main changes occur in the first simulation effort, and multiple experiences improve students’ learning results. Therefore, it is recommended to perform repeated simulation exercises in various courses of the nursing teaching program to achieve optimal learning results, as well as to achieve a high level of competence and satisfaction of the students [22]. Despite the abovementioned benefits of studying through simulation, relatively few studies have examined ways of studying that are done at simulation centers and how they contribute to the competencies of nursing students [23].

Accordingly, our research aims were:

1. The first aim of this study was to evaluate the effect of a fundamental nursing practices simulation (FNPS) on the nursing students’ competencies (clinical care, interpersonal relations, professional development, teaching/coaching, legal/ethical practice, leadership, and critical thinking) and learning satisfaction in three-time points: before, immediately after, and one month after FNPS.
2. The second aim was to detect relations and predictions between the nursing students’ competencies (clinical care, interpersonal relations, professional development, teaching/coaching, legal/ethical practice, leadership, and critical thinking) and learning satisfaction immediately after conducting FNPS and one month after T3.

## 2. Materials and Methods

### 2.1. Study Setting

The study design was a one-group, three-point time repeated measures study.

### 2.2. Setting and Sample

The study site was an academic institution located in the center of Israel. Ninety-three undergraduate nursing students enrolled in the FNPS were convenience sampled. Prerequisite eligibility requirements included successful completion of the first nursing fundamental skills theoretical course (classroom) that occurs at the end of the first year of the nursing program.

### 2.3. Data Collection

The FNPS was composed of six simulation learning days, four hours per day, consisting of medium-fidelity simulators and facilitator-guided reflection sessions for debriefing. As part of the simulation’s practices, students learn and exercise fundamental nursing skills within complex clinical situations. Skills regarding medication, feeding tubes, draining bladders, different types of injections, etc., are exercised through medium-fidelity simulation via mannequins. Afterward, students participated in instruction-guided reflection sessions to debrief and review their performance and identify learning opportunities. As part of the guided reflection sessions, and after the completion of the simulation, students were able to watch a video recording of the simulation team, as well as individual performance. In addition to the regular educational staff, before the simulation, volunteers were selected and underwent pre-training regarding the simulation topics, who served as aides and assisted the educational staff. Following the simulation, students were able to consult with them on matters they were uncertain about. Training days for tutors were held at the beginning of the year and on a voluntary basis. Data collection occurred at three-time points: before (T1), immediately after (T2), and one month after stimulation (T3).

### 2.4. Measures and Instruments

In this study, a questionnaire was composed of three parts:

1. Sociodemographic characteristics, which included information such as background data, age, sex, and more.
2. Competency Inventory for Registered Nurses (CIRN) scale [14], which was used for measuring nursing competency. CIRN was based on the International Council of Nurses Framework of Competencies for the Generalist Nurse. It consists of 58 items divided into seven dimensions: legal/ethical practice, interpersonal relations, leadership, professional development, clinical care, critical thinking/research aptitude, and teaching/coaching. The original reliability for this instrument yielded alpha values ranging from a highest 0.86 for the leadership scale to the lowest 0.79 for professional development [13]. Answers were based on a 5-point Likert-like scale ranging from 0 (not competent) up to 4 (4 = highly competent). One example item was: “Provides culturally-sensitive care”. Cronbach’s for each subscale in this study was as follows: legal/ethical practice, 0.92; interpersonal relations, 0.93; leadership, 0.93; professional development, 0.93; clinical care, 0.94; critical thinking/research aptitude, 0.90; and teaching/coaching, 0.93. Three nursing researchers with very good English confirmed the reliability and authenticity of the translation of the scale from English into Hebrew and vice versa.
3. Learning satisfaction, which was measured with the following question: “What is your level of learning satisfaction while participating in the simulation?” The answer was based on a 10-scale ranging from 1 (not satisfied at all) up to 10 (10 = most highly satisfied) [24].

### 2.5. Data Analysis

Statistical analysis was performed using Statistical Package for the Social Sciences (SPSS TM) version 21.0 (IBM, Chicago, IL, USA). Descriptive statistics, including frequencies, percentages, means, and standard deviations, were conducted to describe the demographic variables. In addition, we performed a one-way repeated measures analysis of variance to examine the effect of FNPS intervention in the three-time points. Post-hoc comparisons were performed using the Bonferroni adjustment for multiple comparisons to evaluate differences in means. Furthermore, correlations and stepwise regressions were conducted for each point in time. In all analyses, the level of significance was set to *p* < 0.05 (two-tailed) using IBM SPSS Statistics (version 25.0).

## 3. Results

### Survey Findings: Sociodemographic Characteristics of the Total Sample

There were 93 nursing students from the university. The mean age of students was 23 years (SD = 4.1; Table 1).

As Table 1 shows, the minority of the students were male (5; 5%), and the majority were female (88; 95%). The majority were single (72; 77%), and the other were married (21; 23%). Most of the students were religious (70; 75%), and only one was orthodox (1; 1%). Most were high school graduates (89; 96%) and others held an undergraduate degree (4; 4%).

To evaluate the effect of FNPS on the nursing students’ competencies (clinical care, interpersonal relations, professional development, teaching/coaching, legal/ethical practice, leadership, and critical thinking) and learning satisfaction in the three-time points, we conducted a one-way repeated measures analysis of variance (Table 2).

A significant effect was found in comparison between the three points in time regarding nursing competencies such as critical thinking, clinical care, interpersonal relations, professional development, teaching/coaching, legal/ethical practice, and leadership (F_(2,93)_ = 58.21, *p* = 0.000, *ηp*^2^ = 0.56), (F_(2,93)_ = 54.03, *p* = 0.00, *ηp*^2^ = 0.54), (F_(2,93)_ = 48.16, *p* = 0.00, *ηp*^2^ = 0.51), (F_(2,93)_ = 53.19, *p* = 0.00, *ηp*^2^ = 0.54), (F_(2,93)_ = 66.52, *p* = 0.00, *ηp*^2^ = 0.59), (F_(2,93)_ = 45.06, *p* = 0.00, *ηp*^2^ = 0.49), and (F_(2,93)_ = 58.63, *p* = 0.00, *ηp*^2^ = 0.56), respectively. All these nursing competencies increased significantly from T1 to T2 and T3, but not from T2 to T3. Moreover, a significant effect was found in the comparison between the three points in time as to nursing students’ learning satisfaction (F_(2,93)_ = 37.48; *p* = 0.000; *ηp*^2^ = 0.45). Nursing students’ learning satisfaction increased significantly from T1 to T2 and T3, but not from T2 to T3. Competencies did not decline between T2 and T3; the effectiveness of the simulation continued over the one-month timeframe.

For detecting relations and predictions between the nursing students’ competencies (clinical care, interpersonal relations, professional development, teaching/coaching, legal/ethical practice, leadership, and critical thinking) and learning satisfaction immediately after conducting FNPS (T2) and one month after (T3), we first conducted Pearson correlations, and then multiple regression stepwise type analysis for T1 and T2.

We found significant relationships between nursing students’ competencies (critical thinking, clinical care, interpersonal relations, professional development, teaching/coaching, legal/ethical practice, and leadership) and learning satisfaction (r = 0.37, *p* < 0.00; r = 0.42, *p* < 0.00; r = 0.36, *p* < 0.00; r = 0.34, *p* < 0.00; r = 0.35, *p* < 0.00; r = 0.44, *p* < 0.00; and r = 0.36, *p* < 0.00), respectively (Table 3).

Table 4 shows relationships between nursing students’ competencies (clinical care, critical thinking, interpersonal relations, professional development, teaching/coaching, legal/ethical practice, and leadership) and satisfaction (r = 0.36, *p* < 0.00; r = 0.37, *p* < 0.00; r = 0.38, *p* < 0.00; r = 0.24, *p* < 0.00; r = 0.38, *p* < 0.00; r = 0.27, *p* < 0.00; and r = 0.36, *p* < 0.00), respectively.

For each point in time, T2 and T3, stepwise analysis regression shows that satisfaction may be explained based on the nursing competency of legal/ethical practice (F_(1,91)_ = 21.42, *p* < 0.00,), (F_(1,91)_ = 16.04, *p* < 0.00,) respectively. The predictive variables explained at T2 were 19% and at T3 were 15% of the variance (Table 5).

Only one nursing competency, legal/ethical practice, explained the variance of learning satisfaction in T2 and T3. The other dimensions were removed from the model by the stepwise analysis (Table 5).

## 4. Discussion

In this study, evidence of exposure to the FNPS intervention helped improve perceived nursing competence and learning satisfaction among nursing students. Regarding the first aim, our results suggest that all nursing competencies increased significantly from T1 to T2 and T3 but not from T2 to T3. Furthermore, nursing students’ learning satisfaction increased significantly from T1 to T2 and T3 but not from T2 to T3. Regarding the second aim, our results suggest that legal/ethical practice could predict satisfaction in T2 and in T3.

The study found that all nursing competencies increased significantly from T1 to T2 and T3. Similar to these results, another study found that FNPS helped to increase nurses’ competencies after simulation learning for the early identification and management of sepsis [25]. Another study conducted cross-sectional research with undergraduate nursing students and found simulation to be a useful tool for the learning process and acquisition of competencies related to emergency situation management [18]. An additional study conducted a quasi-experimental study with 69 nursing students from the Middle East and found that simulation was effective for teaching practical skills for nasogastric tube feeding treatment [26]. In another mixed-method study design, nursing students showed adequate clinical competency following simulation [27].

The current study found that nursing students’ learning satisfaction increased significantly from T1 to T2 and T3. Similar to our results, another study that conducted a randomized control trial found that simulation-based training for in-service nursing education could enhance nurses’ communication performance in clinical practice [28]. Another study demonstrated that nursing students who completed simulated scenarios related to critically ill patients in an emergency room expressed a high level of satisfaction and positive perceptions about clinical simulation sessions [18]. An additional study used patient-care simulation to enhance learners’ satisfaction with learning and self-confidence [29].

Our study results suggest that nursing competency in legal/ethical practice can predict students’ learning satisfaction both immediately after and one month following the simulation. Many studies have shown a relationship between nursing competencies and learning satisfaction regarding simulation training [9,22,30,31,32]. However, after an extensive research literature search, no study was found that examined repeated measures at three points in time for simulation effectiveness related to students’ nursing competency, especially ethical/legal competency and learning satisfaction. One study claims that nursing students are expected to learn and develop independence and competencies such as ethics, safety practices, and a sense of responsibility as they progress in clinical practice. Thus, it is essential to assess the students’ satisfaction with, and effectiveness of, the clinical learning environment to enhance undergraduate nursing students’ education [33]. This study emphasizes the importance of the implementation of ethical/legal competency for nursing students.

This study had a few limitations. First, our study only covered a span of one month, which does not allow for the study of the long-term effectiveness of simulations. Second, the outcomes measured were based on participants’ self-reported surveys. It is possible that with self-reported outcomes, participants do not always answer truthfully. A final limitation was the study’s relatively small sample size.

## 5. Conclusions

Even though it is known that simulation-based learning could enhance nursing competency and learning satisfaction among nurses, this study has revealed that FNPS is effective not just immediately after performing the simulation, but also one month after. We suggest that emphasizing active learning through FNPS may have an essential impact on nursing educational practices and simulation designs. Therefore, it is recommended to implement FNPS in other nursing education-related areas, such as communication skills, geriatric, and mental health fields, since it has a long and wide impact on nursing competency.

Moreover, this study has established that nursing competency of legal/ethical practice can predict students’ learning satisfaction not only immediately after conducting FNPS, but also one month after conducting the simulation. The use of simulations is well-received by nursing students, who maintain a high level of learning satisfaction. It is important to consider learning competency of legal/ethical practice as an educational aim in planning learning simulations. In addition, the novelty of the findings of this study emphasizes the increased importance of learning ethical/legal nursing competency, impacting students’ learning satisfaction not only immediately after simulation, but also one month afterward. Therefore, additional studies are required to validate these important findings for an optimal student clinical learning experience.

## Figures and Tables

**Table 1:** Frequency and percentage of background characteristics (*n* = 93).

Background Characteristic		*n* = 93	
		*n*	%
Sex	Male	5	5
	Female	88	95
Status	Single	72	77
	Married	21	23
Religion	Jewish	89	96
	Other	4	4
Religiosity	Secular	13	14
	Traditional	9	10
	Religious	70	75
	Orthodox	1	1
Education	High school	89	96
	Undergraduates’ degree	4	4

**Table 2:** Means and standard deviations for nursing students’ competencies before (T1), immediately after (T2), and one month after (T3) simulation.

Variables		T1 Mean (SD)	T2 Mean (SD)	T3 Mean (SD)	T1–T2 *p*	T1–T3 *p*	T2–T3 *p*
Nursing students’ competencies	Critical thinking	2.66 (0.94)	3.51 (0.91)	3.65 (0.66)	**	**	0.18
	Clinical care	2.89 (0.92)	3.63 (.84)	3.80 (0.62)	**	**	0.060
	Interpersonal relations	2.94 (0.93)	3.66 (0.88)	3.81 (.63)	**	**	0.17
	Professional development	2.94 (0.97)	3.84 (0.84)	3.90 (0.58)	**	**	0.90
	Teaching/coaching	2.53 (0.98)	3.49 (0.95)	3.66 (0.73)	**	**	0.19
	Legal/ethical practice	3.16 (0.93)	3.99 (0.73)	3.91 (0.64)	**	**	0.65
	Leadership	2.92 (0.91)	3.76 (0.81)	3.85 (0.60)	**	**	0.72
Learning satisfaction		7.15 (13)	8.95 (0.99)	8.81 (1.2)	**	**	0.41

SD—standard deviation; ** *p* < 0.01.

**Table 3:** Pearson correlations between students’ nursing competencies and learning satisfaction immediately after simulation.

Variables	Learning Satisfaction	Critical Thinking	Clinical Care	Interpersonal Relations	Professional Development	Teaching/Coaching	Legal/Ethical Practice	Leadership
Learning satisfaction	1	0.37 **	0.42 **	0.37 **	0.34 **	0.35 **	0.44 **	0.36 **
Critical thinking	0.37 **	1	0.93 **	0.91 **	0.8 **	0.93 **	0.79 **	0.86 **
Clinical care	0.42 **	0.92 **	1	0.92 **	0.82 **	0.93 **	0.85 **	0.92 **
Interpersonal relations	0.36 **	0.91 **	0.93 **	1	0.85 **	0.88 **	0.84 **	0.94 **
Professional development	0.34 **	0.8 **	0.82 **	0.85 **	1	0.75 **	0.78 **	0.86 **
Teaching/coaching	0.35 **	0.93 **	0.93 **	0.88 **	0.75 **	1	0.75 **	0.85 **
Legal/ethical practice	0.44 **	0.79 **	0.85 **	0.84 **	0.78 **	0.75	1	0.86 **
Leadership	0.36 **	0.86 **	0.92 **	0.94 **	0.86 **	0.85 **	0.86 **	1

** *p* < 0.01.

**Table 4:** Pearson correlations between student’s nursing competencies and learning satisfaction one month after simulation.

Variables	Learning Satisfaction	Critical Thinking	Clinical Care	Interpersonal Relations	Professional Development	Teaching/Coaching	Legal/Ethical Practice	Leadership
Learning satisfaction	1	0.36 **	0.36 **	0.37 **	0.38 **	0.24 *	0.38 **	0.27 **
Critical thinking	0.36 **	1	0.93 **	0.9 **	0.76 **	0.91 **	0.82 **	0.84 **
Clinical care	0.36 **	0.93 **	1	0.93 **	0.77 **	0.88 **	0.9 **	0.86 **
Interpersonal relations	0.37 **	0.9 **	0.93 **	1	0.79 **	0.83 **	0.89 **	0.92 **
Professional development	0.38 **	0.76 **	0.77 **	0.79 **	1	0.69 **	0.77 **	0.82 **
Teaching/coaching	0.24 *	0.9 **	0.88 **	0.83 **	0.69 **	1	0.75 **	0.78 **
Legal/ethical practice	0.38 **	0.82 **	0.9 **	0.89 **	0.77 **	0.75 **	1	0.87 **
Leadership	0.27 **	0.84 **	0.86 **	0.92 **	0.82 **	0.78 **	0.87 **	1

* *p* < 0.05; ** *p* < 0.01.

**Table 5:** Multiple regression stepwise type analysis test for predicting satisfaction.

	Model	B	β	t	R^2^
T2	legal/ethical practice	0.59	0.44 **	4.63	0.19
T3	legal/ethical practice	0.61	0.38 **	4.00	0.15

** *p* < 0.01.
